# Assimilation of PSO and SVR into an improved ARIMA model for monthly precipitation forecasting

**DOI:** 10.1038/s41598-024-63046-3

**Published:** 2024-05-27

**Authors:** Laleh Parviz, Mansour Ghorbanpour

**Affiliations:** 1 Faculty of Agriculture, Azarbaijan Shahid Madani University, Tabriz, Iran; 2 Department of Medicinal Plants, Faculty of Agriculture and Natural Resources, Arak University, Arak, 38156-8-8349 Iran

**Keywords:** Improved ARIMA, Monthly, SVR, IARIMA-C-PSO, RPD, Climate sciences, Ecology, Environmental sciences

## Abstract

Precipitation due to its complex nature requires a comprehensive model for forecasting purposes and the efficiency of improved ARIMA (IARIMA) forecasts has been proved relative to the conventional models. This study used two procedures in the structure of IARIMA to obtain accurate monthly precipitation forecasts in four stations located in northern Iran; Bandar Anzali, Rasht, Ramsar, and Babolsar. The first procedure applied support vector regression (SVR) for modeling the statistical characteristics and monthly precipitation of each class, IARIMA-SVR, which improved the evaluation metrics so that the decrease of Theil's coefficient and average relative variance in all stations was 21.14% and 17.06%, respectively. Two approaches are defined in the second procedure which includes a forecast combination (C) scheme, IARIMA-C-particle swarm optimization (PSO), and artificial intelligence technique. Generally, most of the time, IARIMA-C-PSO relative to the other approach, exhibited acceptable results and the accuracy improvement was greater than zero at all stations. Comparing the two procedures, it is found that the capability of IARIMA-C-PSO is higher concerning the IARIMA-SVR, so the decrease in the normalized mean squared error value from IARIMA to IARIMA-SVR and IARIMA-C-PSO is 36.72% and 39.92%, respectively for all stations. The residual predictive deviation (RPD) of IARIMA-C-PSO for all stations is greater than 2, which indicates the high performance of the model. With a comprehensive investigation, the performance of Bandar Anzali station is better than the other stations. By developing an improved ARIMA model, one can achieve a high performance in structure identifying and forecasting of monthly time series which is one of the issues of interest and importance.

## Introduction

The collection of data in successive order at different time points can be defined as time series. Historical time series has the information corresponding to the behavior of data which can be used to find their future trend. Time series analysis in the form of forecasting the future condition of phenomena such as precipitation plays a crucial role in many different scientific fields^1^. The issue to be addressed here is the complexity of hydrological data, which is affected by both the deterministic and stochastic factors^2^. One of the models in this regard is the autoregressive integrated moving average (ARIMA) model in which the future data are calculated based on a linear function of previous data and random errors^3^ and it has been widely used in various forecasting fields^4^ such as the precipitation pattern forecasting using ARIMA in WadiShueib catchment area of Jordan^5^, vegetation temperature condition index (VTCI) forecasting as the remote sensing drought index with ARIMA in the Guanzhong Plain^6^. ARIMA model can be applied for time series with a certain time interval such as a week, or a month which is named as seasonal ARIMA (SARIMA) like rainfall time series forecasting using SARIMA model in the humid region of northeast India^7^, monthly rainfall and temperature by SARIMA model in the South Asian countries^8^, utilization of MODIS time series for leaf area index (LAI) estimation using SARIMA model with periodic length = 46^9^, forecasting of 7Be air concentrations (monthly scale) with SARIMA model in the east coast of Spain^10^, SARIMA model for heat demand forecasting in the district heating system^11^. The SARIMA model is capable of considering the inter-annual variation of each month in the monthly time series, but the problem is losing the information related to the inter-monthly variation of the time series.

To account for both kinds of temporal variations, inter-annual and inter-monthly variations, the improved ARIMA model was proposed with the clustering analysis to classify the monthly time series^2^. The high potential of clustering analysis can be related to the governing pattern of data discovery^12^. In general, an accurate and detailed view of the trend of data can improve the nature of forecasts^13^. A study presented the application of improved ARIMA in Lanzhou, China which led to an increase in forecast accuracy^2^.

Due to the efficiency of improved ARIMA, the development of the model can significantly increase the forecast accuracy, especially at a monthly scale. The focus of this research is on the development of an improved ARIMA using two procedures, where one is related to modeling the relation of each monthly time series with the statistical characteristics of time series, and the other one is related to applying forecast combination concept on the forecasts of different models.

Wang et al. used the linear regression method to model the relationship between monthly series and the statistical characteristics of times series such as maximum values^2^. However, this study proposes the artificial intelligence model instead of the linear regression method. Normally, in many applications, the artificial intelligence models perform better than the regression method. Comparing the performance of multiple regression (MR) and artificial neural network (ANN) for long-term rainfall forecasting showed the higher potential of ANN over MR models in rainfall forecasting using large-scale climate models^14^. The efficiency of the machine learning approach was proved in the study of Pirone et al.^15^ in short-term rainfall forecasting and He et al.^16^ for monthly rainfall prediction. Some structures of models such as ANN, SARIMA, improved ARIMA, and linking improved ARIMA to ANN were used for monthly precipitation forecasting of Rasht and Gorgan stations in Iran. The last structure could remarkably improve the accuracy of forecasts compared to the SARIMA and ANN models^17^.

Many studies showed that information combination or forecast combination are helpful means for forecasting purposes^18–21^. The forecast combination scheme integrates the competing forecasts to produce a composite forecast with superiority to the individual forecasts^21^. Some combination methods are based on the weighting scheme which the groups of calculated forecasts from single models are averaged according to their weights^22^. The greatest weight belongs to the model with the highest efficiency. The weighting schemes have a special mathematical structure and performance such as the error-based methods^23^, the least square regression method^24^, and the differential weighting schemes (DWS)^25,26^.

Some benchmark linear combination methods were used to aggregate multiple forecasts (five single models). The results demonstrated the efficiency of the combination schemes compared to the single models^27^. Another way to determine the weights of the single models is the definition of the problem as an optimization algorithm^28^. The combination approach was defined for agricultural commodity price forecasting with ARIMA, support vector regression (SVR), recurrent neural network (RNN), gated recurrent neural network (GRU), and long-short-term memory neural network (LSTM) as the forecasting models. The optimized weighting vector was determined by an artificial bee colony (ABC) algorithm with high performance^29^. Another study applied ABC to find the weights of forecast combination schemes from linear regression, ANN, and SVR models^21^. To forecast wind speed, five single models are introduced and for the combination of mentioned models, some optimization strategies were proposed such as particle swarm optimization (PSO) and genetic algorithm (GA). A significant difference was not observed between the two optimization methods^30^. Therefore, considering the efficiency of the forecast combination scheme, its assimilation with an improved ARIMA seems to be a suitable option for upgrading precipitation forecasts.

Due to the importance of forecasting in various fields, especially in the case of precipitation with its complex nature, an efficient model or developed model is needed^31,32^. Therefore, this study attempts to increase the accuracy of the improved ARIMA model for monthly precipitation forecasting because of the superiority of this model over existing models such as SARIMA. In this regard, the emphasis is on the modeling between monthly precipitation series and statistical characteristics of each cluster in which the artificial intelligence model was implemented compared to the original model of improved ARIMA (linear regression, LR). In addition to improving the modeling procedure in the improved ARIMA structure, the forecast combination scheme was applied to this model for deriving the efficient information of each forecasting model, i.e. SVR and LR, until the improved ARIMA model is developed. One of the approaches of the forecast combination schemes is the application of the optimization method, PSO, to find the optimal weights of the models.

## Material and methods

### Case study

**Figure 1:**
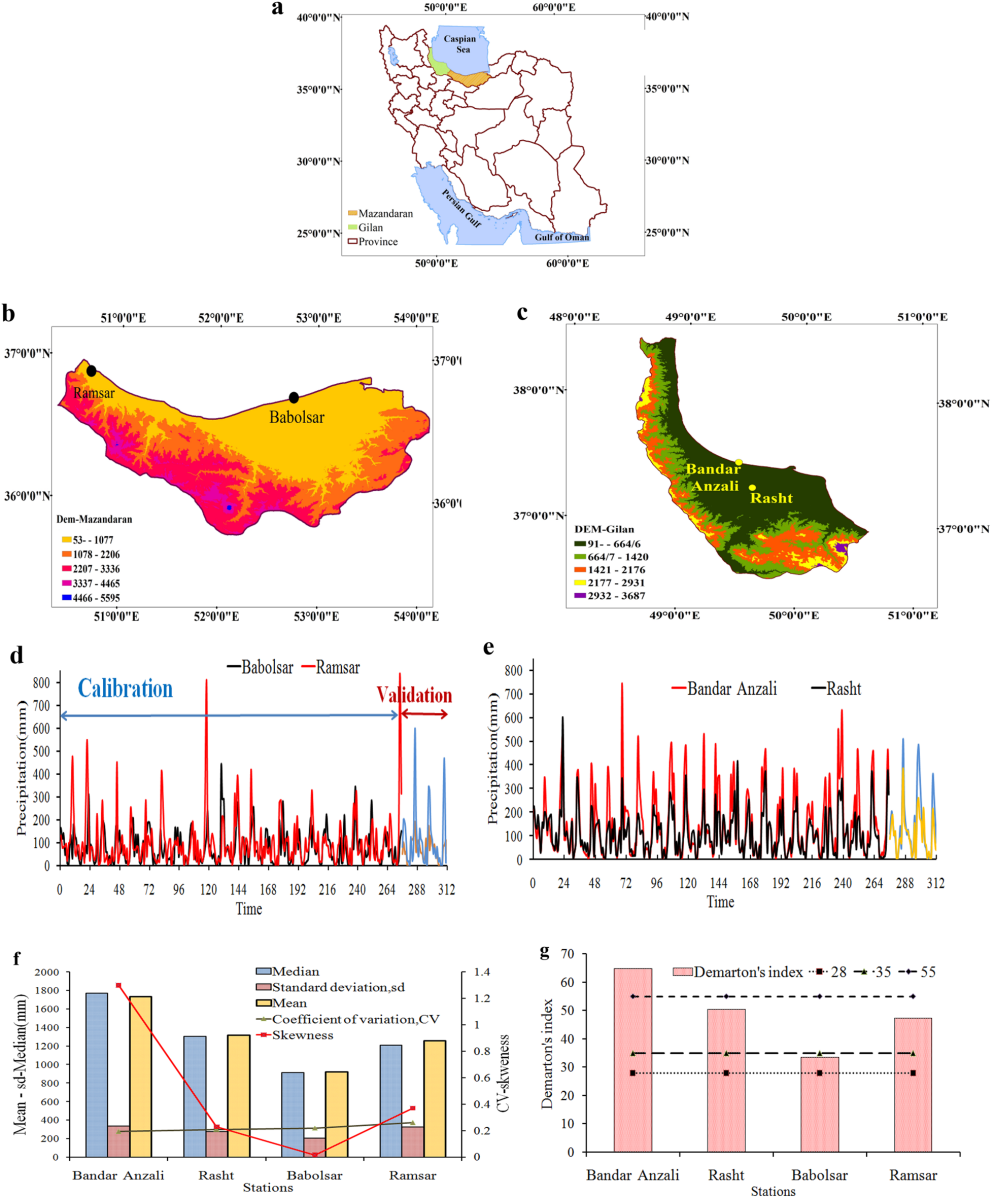
(**a**) The location of studied provinces in Iran, (**b**,**c**) stations in each province, (**d**,**e**) precipitation time series in all stations, (**f**) statistical characteristic of time series, and (**g**) climate of each station based on De Marttone climate classification.

Iran is divided into six main river basin regions the Caspian Sea basin located in the north of Iran with a 174,000 km^2^ area is one of them. The precipitation data of this study belong to the stations of the Caspian Sea basin. The used data corresponded to the Rasht, Bandar Anzali, Ramsar, and Babolsar stations, where Rasht and Bandar Anzali are in Gilan province and the last two stations are in Mazandaran province. The location of stations in each province and the location of provinces in Iran are shown in Fig. 1a–c. The period of study is from 1992 to 2020, where the 1992–2014 period was used for the calibration process. The monthly time series in the calibration and validation periods are drawn in Fig. 1d,e. The statistical characteristics of the time series are given in Fig. 1f. The average annual precipitation at all the stations was equal to 1309.2 mm, where the highest precipitation belonged to the Bandar Anzali station. The maximum and minimum values of the variation coefficient corresponded to Ramsar and Bandar Anzali stations, respectively. The climate of stations is classified based on the De Marttone^33^ index, in which the climate of Babolsar station is humid, that of Ramsar and Rasht stations is very humid and Bandar Anzali station is extremely humid and the ranges of the index are given in Fig. 1g. Based on Koppen^34^ climate classification, the climate of all stations is humid, Cfa.

### Improved ARIMA model

The improved ARIMA (IARIMA) model attempts to consider the inter-annual and inter-monthly variations, simultaneously, to forecast the monthly time series, which is not included in the previous SARIMA model. The improved ARIMA model consists of some proceedings such as:

1. The first step applies the clustering analysis to classify the monthly time series according to their specifications^2^. The clustering algorithm involves the collection of similar data in a group that is different from the data classified in the other groups. Up to now, some clustering methods have been proposed the hierarchical clustering is one of them^35,36^. Hierarchical clustering is one of the important methods used in the clustering field. The method attempts to separate data at distinct levels to constitute a clustering structure in the form of a tree^37^. The mathematical form of the first step can be expressed as Eq. (1):

1 $$\begin{array}{*{20}c} {{}_{{\arg { }\min {\text{ dis }}\left( {{\text{C}}_{{\text{I}}} ,{\text{x}}} \right)^{2} { }}}^{{{\text{clustring}}}} \;({\text{P}}_{{\text{i}}} )} & {{\text{to}}\;{\text{the }}\;{\text{kth}}\;{\text{ groups}}} & {{\text{P}}_{{\text{i}}} :{\text{i}} = 1, \ldots ,12} \\ {{\text{for }}\;{\text{example}}:} & {{\text{class}}\;1:{\text{p}}_{1} ,{\text{p}}_{2} , \ldots ,} & {{\text{class }}\;{\text{k}}:{\text{p}}_{5} ,{\text{p}}_{6} ,{\text{ p}}_{9} } \\ \end{array}$$

where dist () is the Euclidean distance and c_i_ is the centroid of each cluster.

2. At this step, the statistical characteristics of each cluster such as maximum, minimum, and truncated mean are calculated.

3. Determining the relationship between the statistical characteristics of each cluster with the monthly time series of that cluster. Linear regression model (LRM) was implemented in the proposed improved ARIMA model by Wang et al.^2^ where the statistical characteristics and monthly time series of each cluster were introduced as the independent and dependent variables, respectively. This study incorporates both the linear regression and SVR (IARIMA-SVR) methods for this step (Eq. 2).

2 $$\begin{aligned} & {\text{for}}\;{\text{cluster}}\;{\text{k}}\quad {\text{y}}_{{{\text{i}} = {}_{{{\text{LRM}}}}^{{\mathbf{f}}} \left( {{\text{y}}_{{{\text{k}},{\text{max}}}} {\text{y}}_{{{\text{k}},{\text{min}},}} {\text{y}}_{{{\text{k}},{\text{mean}}}} } \right)}} \;{\text{i}} = 1:{\text{number}}\;{\text{of }}\;{\text{months}}\;{\text{in}}\;{\text{cluster}}\;{\text{k}} \\ & {\text{y}}_{{{\text{i}} = {}_{{{\text{SVR}}}}^{{\text{f}}} \left( {{\text{y}}_{{{\text{k}},{\text{max}}}} {\text{y}}_{{{\text{k}},{\text{min}},}} {\text{y}}_{{{\text{k}},{\text{mean}}}} } \right)}} \quad {\text{i}} = 1:{\text{number}}\;{\text{of }}\;{\text{months }}\;{\text{in}}\;{\text{cluster}} \\ \end{aligned}$$

where y_k,max_,y_k,min_, y_k,mean_ are the maximum, minimum, and average of time series in cluster k.

4. Construct the ARIMA models to forecast the statistical characteristics of each cluster in the desired period. ARIMA is one of the popular models in the field of time series analysis. The main structure of ARIMA can be described in Eq. (3).

3 $$\begin{aligned} \varphi \left( B \right)\nabla^{d} z_{t} & = \theta \left( B \right)a_{t} \\ \varphi \left( B \right) & = 1 - \varphi_{1} B - \varphi_{2} B^{2} - \cdots - \varphi_{P} B^{P} \\ \theta \left( B \right) & = 1 - \theta_{1} B - \theta_{2} B^{2} - \cdots - \theta_{q} B^{P} \\ \end{aligned}$$

where B is the backward shift operator, p,q: orders of autoregressive and moving average parts^38^.

5. Replace the forecasted statistical characteristics of the fourth step with the third step to get the monthly precipitation time series in the desired period^2^.

**Figure 2:**
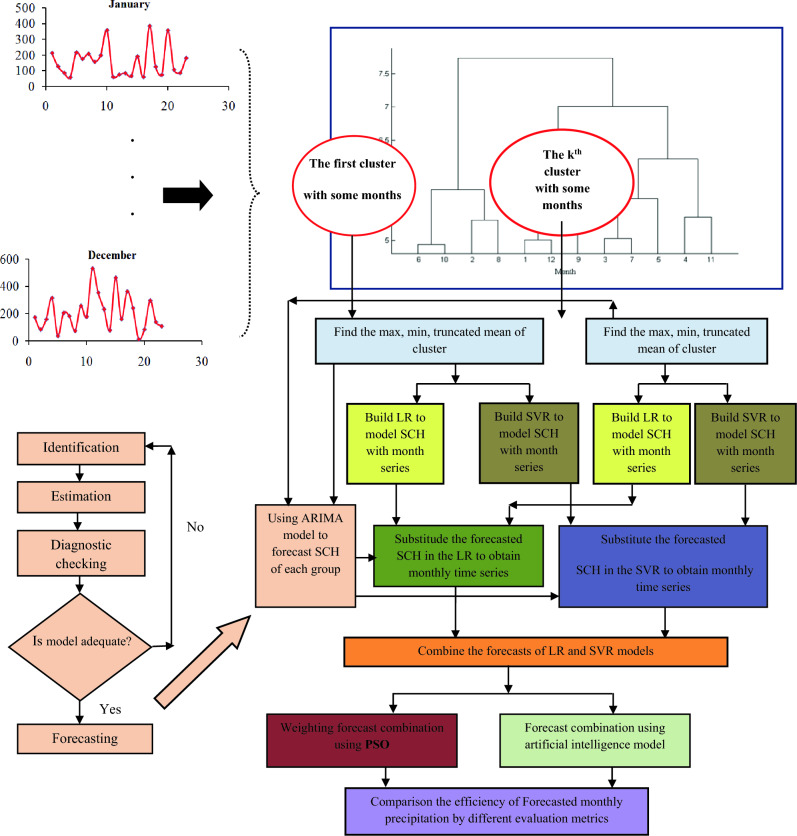
The composition of the developed ARIMA model, IARIMA model implementation by clustering analysis, and then SVR application to model the statistical characteristic (SCH) and monthly series and the final process with the utilization of forecast combination scheme.

The focus of this study is on the improved ARIMA development which is conducted using two procedures. The first procedure is concerned with the relationship of the statistical characteristics of each class with associated monthly time series by SVR instead of LRM, and the second procedure deals with applying the forecast combination scheme to combine the forecasts of single models. The process of research is depicted in Fig. 2 and their codes are in the Supplementary file S1.

### Support vector regression

Support vector regression is one of the machine learning methods which is based on the statistical learning theory^39^. SVR is the adaptation of the SVM concept for regression problems. The efficiency of SVR has been proven in many fields^40–42^. The regression model can be defined as f (x_i_) = **w**^T^**x**_i_ + b. One of the objectives in this case is complexity reduction by minimizing the Euclidean norm of **w**. When the dual form of this problem is derived, the nonlinear version of SVR can be collected. In this case, the kernel function k (**x**_**i**_, **xs**) can be replaced with the dot products which can map the observations to a higher dimensional feature space. Therefore, the kernel-based SVR formulation can be described as an optimization problem in Eq. (4).

4 $$\begin{gathered} {}_{{\alpha ,\alpha^{*} }}^{max} \mathop \sum \limits_{i = 1}^{m} (y_{i} \left( {\alpha_{i} - \alpha_{i}^{*} } \right) - \varepsilon \left( {\alpha_{i} + \alpha_{i}^{*} } \right)) - \frac{1}{2}\mathop \sum \limits_{i,s = 1}^{m} \left( {\alpha_{i} - \alpha_{i}^{*} } \right)\left( {\alpha_{s} - \alpha_{s}^{*} } \right)K\left( {x_{i} ,x_{s} } \right) \hfill \\ subject to \hfill \\ \mathop \sum \limits_{i = 1}^{m} \left( {\alpha_{i} - \alpha_{i}^{*} } \right) = 0 \hfill \\ 0 \le \alpha_{i} ,\alpha_{i}^{*} \le C, i = 1, \ldots ,m \hfill \\ \end{gathered}$$

Linear, Radial basis function or Gaussian are among the most popular kernel functions where their formulation are given in Eqs. (5 and 6), respectively^43^.

5 $$k\left( {x_{i} ,x_{s} } \right) = X_{i}^{T} X_{s}$$

6 $$k\left( {x_{i} ,x_{s} } \right) = \exp \left( { - \gamma x_{i} - x_{s}^{2} } \right), \gamma > 0$$

Among the issues that might affect the efficiency of SVR, one could refer to the type of kernel function and the value of the penalty parameter. The penalty parameter can control the tolerance of the systematic outliers^44^.

### Forecast combination

In recent years, the acceptable performance of some models such as stochastic models; SARIMA^45^, and artificial intelligence techniques such as ANN^46^, group method of data handling (GMDH)^47^, SVR^48^ has been conclusively observed in forecasting issues in the various scientific fields. Despite the acceptability of single forecasting models, they are associated with low precision risks and instability^49,50^. To reduce the deficiency of single models, the forecast combination concept was proposed by Bates and Granger^51^ to obtain the most accurate forecasts. The forecast combination scheme takes advantage of different single-model forecasts^52^. The efficiency of the forecast combination scheme has been proven in many research works^50,53,54^. The easiest type of combination scheme can be a simple average. Various forecast combination schemes are the combination with variance–covariance of single models^51^, combination with regression on single models^55^, and weighting method^56^. The last-mentioned scheme is based on the appropriate weight allocation to the single models according to their efficiency. Eventually, the forecast combination can be obtained according to Eq. (7).

7 $$\Phi \left( {\hat{y}} \right) = w^{T} \cdot \hat{y}$$

where w is the obtained weights (m by 1), m is the number of models.

Some methods which can help with finding the weights of single models are the error-based methods^23^, and the differential weighting schemes (DWS)^25,26^. Also, some metaheuristic methods have been proposed to find the optimized weights of the single models such as GA^57^, and simulated annealing approach (SA)^58^. Therefore, the issue statement as an optimization problem needs the definition of objective function and constraints. The aim is to minimize the error between observed and forecasted (from different single models) values. In this case, the structure of the optimization problem can be defined as given by Eq. (8).

$$Minimize:$$

8 $$error=\sum_{i=1}^{n}({y}_{io}-{y}_{i\text{f}})=\sum_{i=1}^{n}\left[{y}_{io}-\sum_{j=1}^{m}({w}_{j}\times \widehat{{y}_{i,j}})\right]$$

$$\sum_{i=1}^{m}{w}_{i=1}$$

where w_i_ denotes the weights of single models, y_io_ denotes the observed values, y_if_ is the forecasted values from the weighting method, and m is the number of single models.

The structure of error in Eq. (8) can be defined in different forms such as the mean absolute error (MAE). This study incorporates the PSO algorithm with improved ARIMA (IARIMA-C-PSO) to combine the forecasts of two models. PSO relative to other optimization algorithms like GA, is simpler and easier to implement. The dominance of the algorithm is on the individual and global optimum which it does not need for too many parameters adjustment. Another advantage of PSO is simple calculation and convergence with a high rate^30^.

### Particle swarm optimization

One of the most effective optimization algorithms with bird flocking behavior is particle swarm optimization^59^. According to previous studies, PSO is very competitive in optimization problems. The social interaction of this algorithm can provide aggregated smart behavior^60^. The solution of PSO is considered as a particle that movies in a hyper-space^59^. Two velocity components are involved with the movement of a swarm of birds with two tendencies, the best global and optimal local entities^60^. The ith particle consists of two characteristics, the position and velocity vector. The learning of each particle is possible from its previous best personal and neighborhood positions designated by *PBEST* and *NBEST*, respectively. The particle position and velocity are determined through adjustment of each particle concerning the *BPEST* and *NBEST*as given in Eq. (9 and 10), respectively.

9 $${x}_{i}^{d}\left(t+1\right)={x}_{i}^{d}\left(t\right)+{v}_{i}^{d}\left(t+1\right)$$

10 $${v}_{i}^{d}\left(t+1\right)=\omega \times {v}_{i}^{d}\left(t\right)+{c}_{1}\times {rand1}_{i}^{d}\left(t\right)\times \left({pbest}_{i}^{d}\left(t\right)-{x}_{i}^{d}\left(t\right)\right)+{c}_{2}\times {rand2}_{i}^{d}\left(t\right)\times \left({nbest}_{i}^{d}\left(t\right)-{x}_{i}^{d}\left(t\right)\right)$$

where ω is the inertia weight, c_1,_ and c_2_ are the acceleration coefficients, d is the dimension of problem,$rand1_{i}^{d}$ and $rand2_{i}^{d}$ are two uniformly distributed random numbers between 0 and 1, t is the number of iterations^59^.

### An artificial intelligence technique for forecast combination

Another approach to combine the forecasts of SVR and linear regression in the improved ARIMA is the artificial intelligence model where in this study, SVR was utilized (IARIMA-C-SVR).

A sensitivity analysis was carried out based on the type of kernel function and penalty parameter. The forecasts of linear regression and SVR are used as the inputs of SVR. Equation 11 shows the SVR with a linear kernel function.

11 $$\hat{y}_{{n,m =\phantom{a}_{{f\left( {x_{i} } \right) = W^{T} x_{i} + b,k\left( {x_{i} ,x_{s} } \right) = X_{i}^{T} X_{s} }}^{f} (\hat{y}_{n,m}^{LR} ,\hat{y}_{n,m}^{SVR} ) \quad m = 1, \ldots ,12 }}^{c}$$

where n and m denote year and month, respectively.

To investigate the impact of development procedures on the improved ARIMA efficiency, some evaluation metrics are employed which are Variance Accounted For (VAF), Average Relative Variance (ARV), Normalized Mean Squared Error (NMSE), Prediction Of Change In Direction (POCID), RMSE, Relative Root Mean Square Error (RRMSE), Theil's coefficient (UI and UII), modified index of agreement (d), Residual Predictive Deviation (RPD)where their corresponding Eq. are given in Eqs. (12, 13, 14, 15, 16, 17). A large number of evaluation metrics provide a comprehensive assessment of the efficiency of models.

12 $$NMSE=\frac{\sum_{t=1}^{n}{({\widehat{y}}_{t-}{y}_{t})}^{2}}{\sum_{t=1}^{n}{({y}_{t-}{y}_{t+1})}^{2}}$$

13 $$RV=\frac{1}{n}\frac{\sum_{t=1}^{n}{({\widehat{y}}_{t-}{y}_{t})}^{2}}{\sum_{t=1}^{n}{({\widehat{y}}_{t-}\overline{y })}^{2}}$$

14 $$POCID=\frac{100}{N}\sum_{t=1}^{n}{D}_{t}, {D}_{t}=\left\{0\begin{array}{c}1 \left({y}_{t-}{y}_{t-1}\right)\left({\widehat{y}}_{t}-{\widehat{y}}_{t-1}\right)\\ otherwise\\ \end{array}\right.$$

15 $$UI=\frac{{\left[\sum_{t=1}^{n}{({\widehat{y}}_{t-}{y}_{t})}^{2}\right]}^{0.5}}{{\left[\sum_{t=1}^{n}{({y}_{t})}^{2}\right]}^{0.5}+{\left[\sum_{t=1}^{n}{({\widehat{y}}_{t})}^{2}\right]}^{0.5}} UII=\frac{{\left[\sum_{t=1}^{n}{({\widehat{y}}_{t-}{y}_{t})}^{2}\right]}^{0.5}}{{\left[\sum_{t=1}^{n}{({y}_{t})}^{2}\right]}^{0.5}}$$

16 $$RMSE=\frac{1}{n}\sqrt{\sum_{t=1}^{n}{({\widehat{y}}_{t-}{y}_{t})}^{2}}, RRMSE=\frac{RMSE}{\overline{O} }, RPD=\frac{SD}{RMSE}$$

17 $$d=1-\frac{\sum_{t=1}^{n}\left|{y}_{i-}{\widehat{y}}_{i}\right|}{\sum_{t=1}^{n}\left(\left|{y}_{i-}\overline{y }\right|+\left|{\widehat{y}}_{t-}\overline{y }\right|\right)}$$

where $\hat{y}_{i}$ is the forecasted value, y_i_ is the observed value,$\overline{y}$ is the mean of the observed value.

The NMSE values greater than one indicate that forecasts are not good. For comparing the differences between the forecast and the average of the entire sequence, ARV can be used. The ARV values less than 1 indicate a useful forecast. Knowing about the accuracy of the directional change prediction is possible with POCID^61^. The minimum values of RMSE, RRMSE, UI, and UII indicate the desired values^62^. The range of d is between 0 and 1 and a greater value is indicative of the high performance of the model. RPD values less than 1.4 indicate poor performance and greater than 2 represent high performance^63^. The higher values of VAF are indicative of forecast improvement^64^.

## Results

In this section, the results of improved ARIMA and the development of improved ARIMA are investigated using the monthly precipitation series at the four stations. The development of the model has been conducted using two procedures, The first is to use one of the artificial intelligence techniques, SVR, in the modeling process of IARIMA, and the other is related to the forecast combination scheme applying to the results of IARIMA (SVR and LR). In this case, the comprehensive evaluation metrics were applied for the evaluation of the model's performance and its improvement detection.

### Improved ARIMA

**Figure 3:**
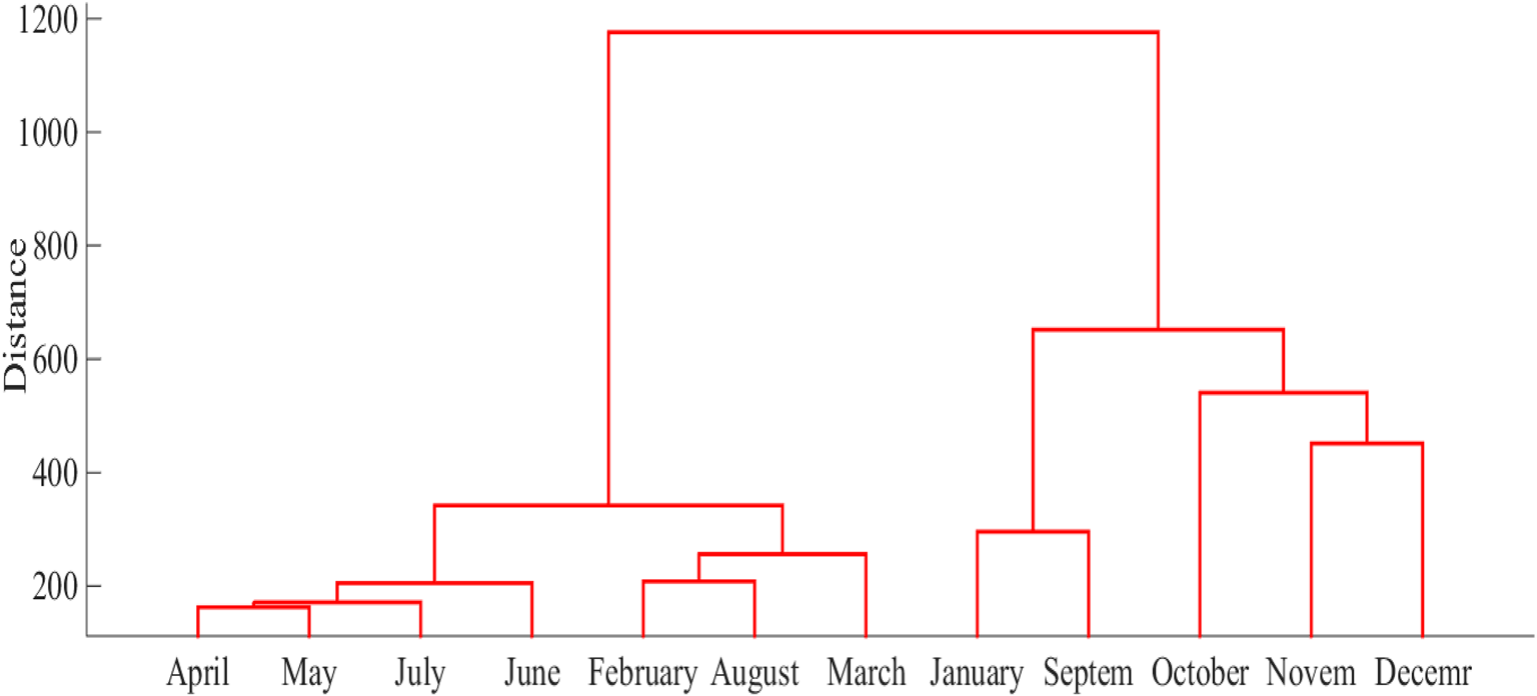
Dendrogram of monthly precipitation series in Babolsar station, the Euclidean distance is shown by the y-axis.

The first step of improved ARIMA is clustering analysis to classify the monthly data with similar characteristics. We applied hierarchical clustering with the Euclidean distance to measure the distance for clustering analysis and the ward method. Among the methods of hierarchical clustering, the performance of the ward method is desirable as it could minimize the variance of elements in each cluster^65^. Representation of cluster similarity matrix is possible with a dendrogram as shown in Fig. 3. It helps with finding the months with similar characteristics and then selecting them in a group. The results of the classification are given in Table 1.

**Table 1:** The results of monthly precipitation clustering with hierarchical clustering.

Bandar Anzali		Babolsar		Ramsar		Rasht	
Class1	April	Class1	February	Class1	January	Class1	April
	May		March		May		May
	July		August		April		June
Class 2	February	Class 2	April		August		July
	March		May		June		August
	June		June		July	Class 2	October
	August		July	Class2	February		November
Class 3	January	Class 3	January		March	Class 3	January
	October		September		December		February
	November	Class 4	October	Class 3	September		March
Class 4	September		November		October		September
	December		December		November		December

In Table 1, the number of classes corresponding to Bandar Anzali, and Babolsarstations is four, and that of Ramsar, and Rasht stations is three. The noteworthy point in Table 1 is that all the stations are in the same area in terms of climate, but the numbers of classes are different. Therefore, clustering with the precise method helps with grouping the months with the same similarity. The next step of IARIMA after the clustering analysis, is finding the statistical characteristics (maximum, minimum, and truncated mean) of each class. The variation coefficient of statistical characteristics related to each class was calculated and the results are given in Table 2. The maximum values of the variation coefficient in all the classes and at all stations in Table 2 are related to the minimum series, except for class 3 at Ramsar station which is related to the truncated mean time series. Some variation coefficient values of statistical characteristics are higher than one, such as the first and second classes at the Bandar Anzali and Rasht stations, respectively. The other step of IARIMA is to construct the linear regression models between the statistical characteristics of each class and the monthly time series. Next, the AIRIMA model is used for the statistical characteristics series forecasting in the validation period. The structure of ARIMA models is given in Table 3 for all the stations.

**Table 2:** The variation coefficient of statistical characteristic related to each class.

Station	Class	Maximum	Minimum	Truncated mean	Station	Maximum	Minimum	Truncated mean
Bandar Anzali	1	0.54	**1.16**	0.63	Babolsar	0.41	**0.85**	0.45
	2	0.62	**0.88**	0.51		0.51	**0.86**	0.62
	3	0.30	**0.60**	0.45		0.33	**0.55**	0.38
	4	0.45	**0.58**	0.43		0.37	**0.66**	0.38
Rasht	1	0.37	**0.68**	0.49	Ramsar	0.30	**0.90**	0.40
	2	0.44	**1.17**	0.63		0.46	**0.68**	0.42
	3	0.43	**0.49**	0.41		0.59	0.59	**0.63**

**Table 3:** The ARIMA model structure for forecasting the statistical characteristic of each class.

Station	Class	Maximum	Minimum	Truncated mean	Station	Maximum	Minimum	Truncated mean
Bandar Anzali	1	ARIMA (1,1,4)	ARIMA (1,0,5)	ARIMA (2,0,4)	Babolsar	ARIMA (3,0,3)	ARIMA (0,0,3)	ARIMA (0,0,3)
	2	ARIMA (0,0,5)	ARIMA (1,0,3)	ARIMA (4,0,2)		ARIMA (5,0,3)	ARIMA (0,0,5)	ARIMA (1,0,2)
	3	ARIMA (5,0,2)	ARIMA (2,0,4)	ARIMA (3,0,3)		ARIMA (0,0,4)	ARIMA (0,0,4)	ARIMA (0,0,4)
	4	ARIMA (0,0,5)	ARIMA (5,1,2)	ARIMA (0,0,4)		ARIMA (0,0,5)	ARIMA (0,0,1)	ARIMA (1,0,5)
Rasht	1	ARIMA (4,0,3)	ARIMA (0,0,5)	ARIMA (5,0,4)	Ramsar	ARIMA (0,0,3)	ARIMA (0,0,3)	ARIMA (4,0,5)
	2	ARIMA (4,0,3)	ARIMA (3,1,2)	ARIMA (5,0,2)		ARIMA (1,1,1)	ARIMA (4,1,2)	ARIMA (5,0,1)
	3	ARIMA (2,0,4)	ARIMA (0,0,5)	ARIMA (4,0,2)		ARIMA (2,0,2)	ARIMA (4,1,4)	ARIMA (1,0,3)

**Figure 4:**
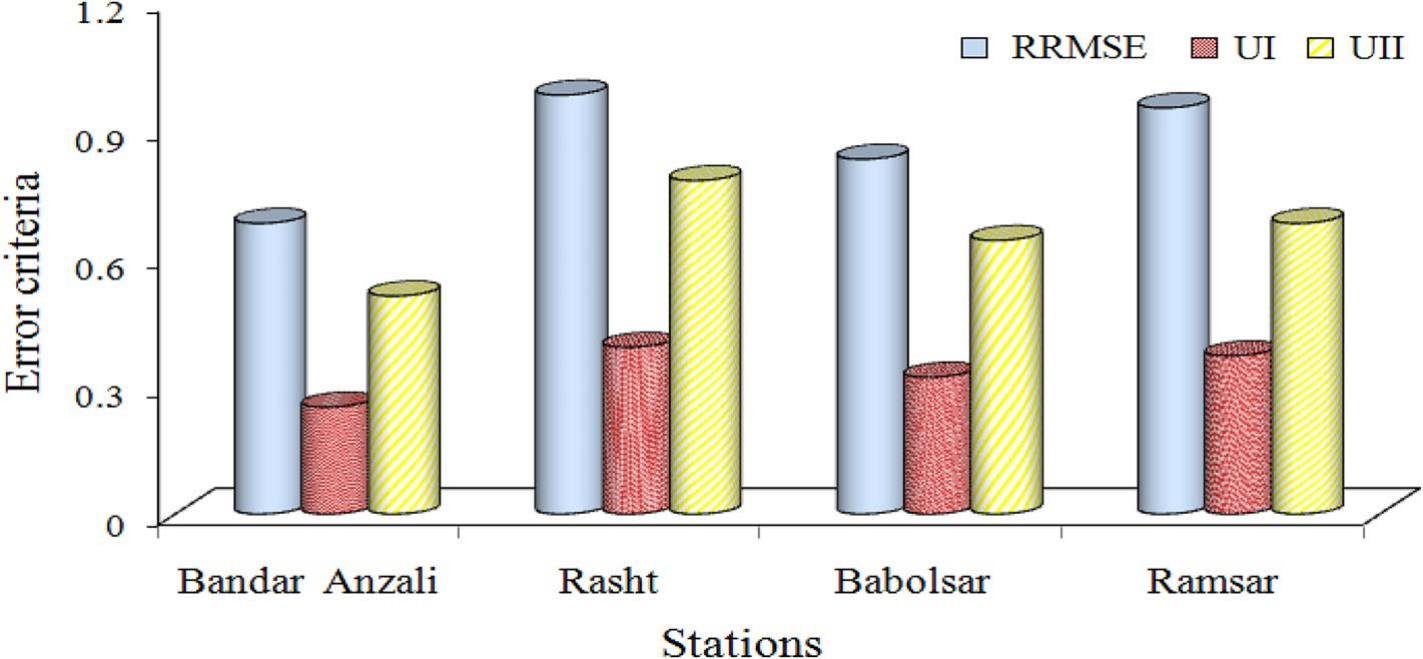
Evaluation metrics, error criteria, for assessment of the monthly precipitation forecasting of IARIMA at four stations in the validation period.

According to Table 3, the structure of the ARIMA model is different in terms of the statistical characteristics of the stations and this structure is distinct for the maximum, minimum, and truncated mean time series of each class at the station. For example, the differencing degree of the maximum time series at the Bandar Anzali station for the first class is 1 and for the truncated mean series is 0. In the end, the forecasted statistical characteristics are substituted in the determined linear regression models, and then the monthly time series are obtained for the 2015–2020 period. The evaluation of the forecasted monthly time series is given in Fig. 4 for all the stations.

The order of error criteria from minimum to maximum values at all the stations (as shown in Fig. 4) is Bandar Anzali, Babolsar, Ramsar, and Rasht (RRMSE_BandarAnzali_ = 0.68, RRMSE_Rasht_ = 0.98, RRMSE_Babolsar_ = 0.83 and RRMSE_Ramsar_ = 0.95). If the average of error criteria is calculated at each station, the deceased average of error criteria from Ramsar, Babolsar, and Rasht to Bandar Anzali stations is equal to 28%, 19.5%, and 33.02% respectively, with the maximum value belonging to Rasht station. The maximum correlation coefficient between the forecasted and observed monthly time series belonged to Bandar Anzali station (0.7).

### Implement of improved ARIMA with SVR

**Figure 5:**
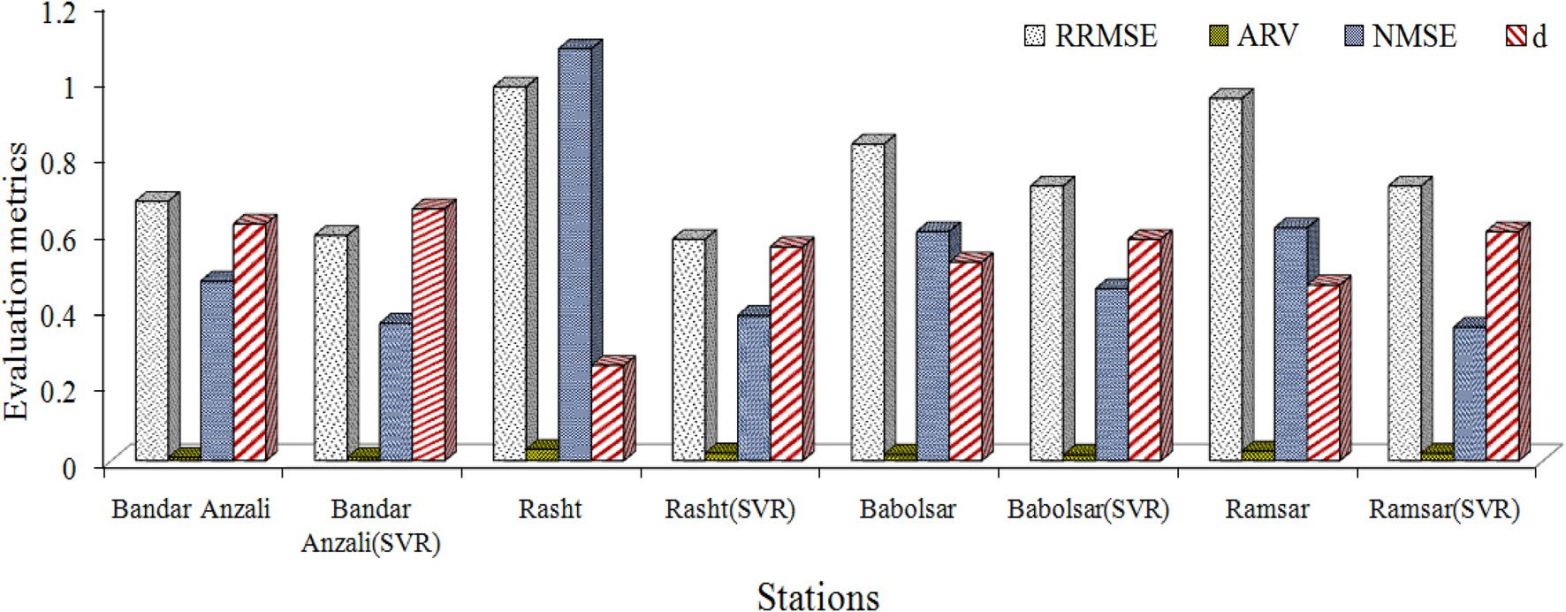
Comparison of the efficiency of IARIMA-SVR against IARIMA at four stations for monthly precipitation forecasting with different evaluation metrics.

The original improved ARIMA used linear regression to model the relation between monthly time series and statistical characteristics of each class. This study attempts to perform the mentioned modeling with SVR. One of the problems that can impact the SVR efficiency is the precise determination of the Kernel function and the values of penalty parameter and it is possible by application of the sensitivity analysis, for example, the decrease of RMSE from Gaussian to sigmoid types of kernel function (penalty parameter = 1) was 48.56% at Bandar Anzali station (first month). Also, the decrease of RMSE using the Gaussian kernel function (with the penalty parameter values of 0.25 to 1) was 17.95% at Ramsar station (fifth month). The kernel functions of 12 months at Bandar Anzali station were Gaussian, sigmoid, sigmoid, Gaussian, Gaussian, Gaussian, sigmoid, linear, linear, linear, Gaussian, Gaussian, respectively. Furthermore, all the kernel functions at Rasht station were Gaussian except for the fourth and sixth months, which were of the sigmoid kernel function. The kernel functions at Ramsar station were sigmoid, sigmoid, sigmoid, Gaussian, Gaussian, sigmoid, sigmoid, Gaussian, sigmoid, sigmoid, sigmoid, and Gaussian. The kernel functions at Babolsar station were Gaussian, Gaussian, linear, sigmoid, Gaussian, sigmoid, Gaussian, Gaussian, Gaussian, Gaussian, Gaussian, and Sigmoid. Generally, the number of Gaussian kernel functions was higher than the sigmoid type at Rasht, Babolsar, and Bandar Anzali stations, and the number of sigmoid kernel functions at Ramsar station was higher than the Gaussian. The effect of improved ARIMA implementation using SVR was investigated by some evaluation metrics which their values are given in Fig. 5.

**Figure 6:**
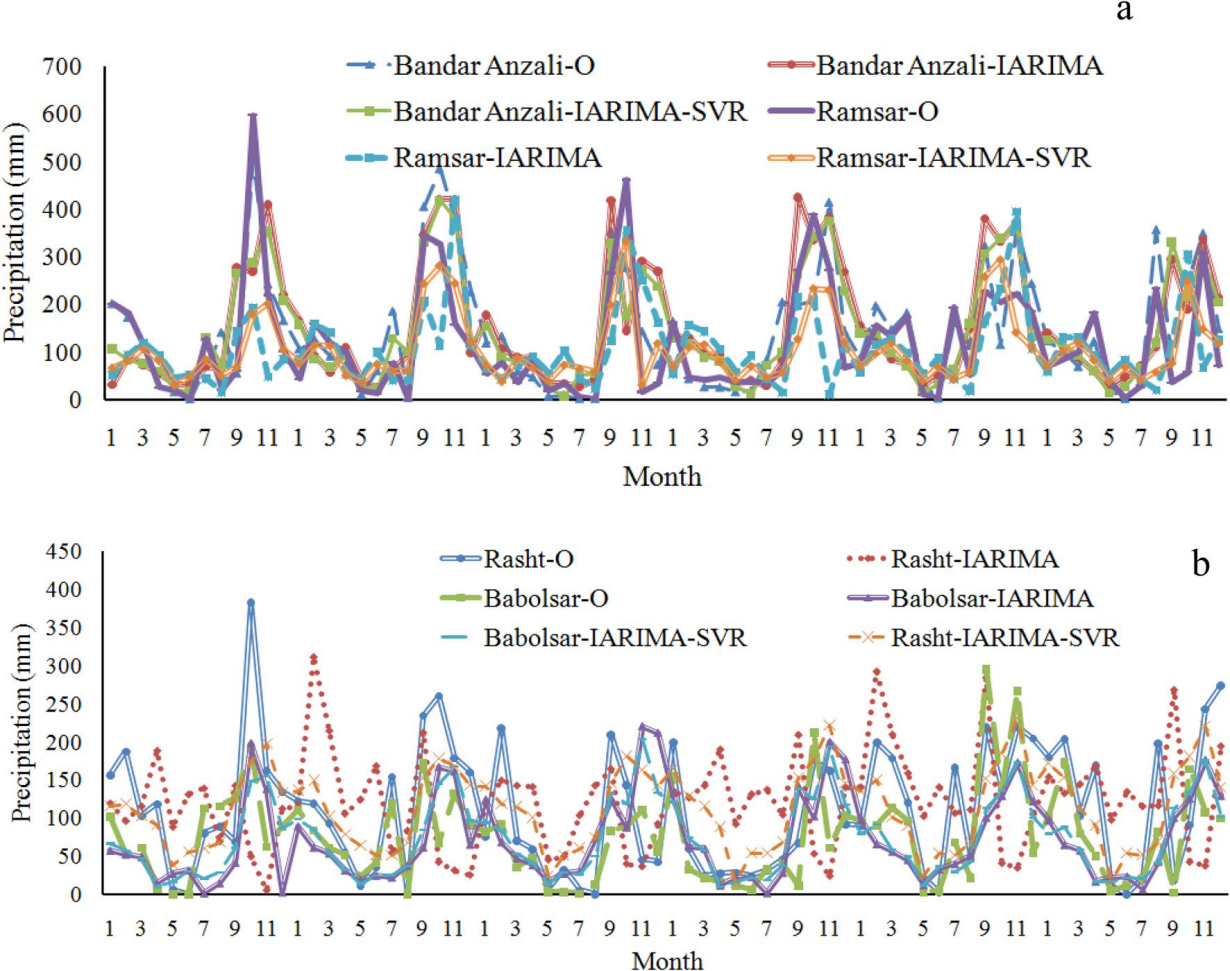
The observed and forecasted (IARIMA and IARIMA-SVR) monthly precipitation time series in the validation period for Bandar Anzali and Ramsar (**a**), Rasht and Babolsar (**b**) stations, O stands observation.

Figure 5 shows the evaluation metrics improvement by implementing the improved ARIMA with the SVR. The decrease of RRMSE using the improved ARIMA concerning IARIMA-SVR was 13.23%, 40.81%, 13.25%, and 24.21%, also the decrease of ARV was 4.7%, 33.33%, 6.25% and 24 and decrease of NMSE was 23.4%, 64.81%, 25% and 42.62% at Bandar Anzali, Rasht, Babolsar and Ramsar stations, respectively. The maximum and minimum of decreased RRMSE values by implementing the improved ARIMA concerning IARIMA- SVR corresponded to Rasht and Bandar Anzali stations, respectively. IARIMA-SVR can increase the values of d relative to IARIMA, such as the increased value of d at Ramsar station concerning IARIMA-SVR was 30.43%. The maximum value of d belonged to Bandar Anzali station. The minimum values of ARV and NMSE of the IARIMA-SVR model belong to Bandar Anzali and Ramsar stations, respectively. Also, it is noteworthy that the difference between the NMSE values corresponding to Bandar Anzali and Ramsarstations is low. For a better understanding of the variation related to the forecasts obtained by the application of improved ARIMA and IARIMA-SVR against the observation data, the graph of monthly precipitation helps a lot (Fig. 6).

**Figure 7:**
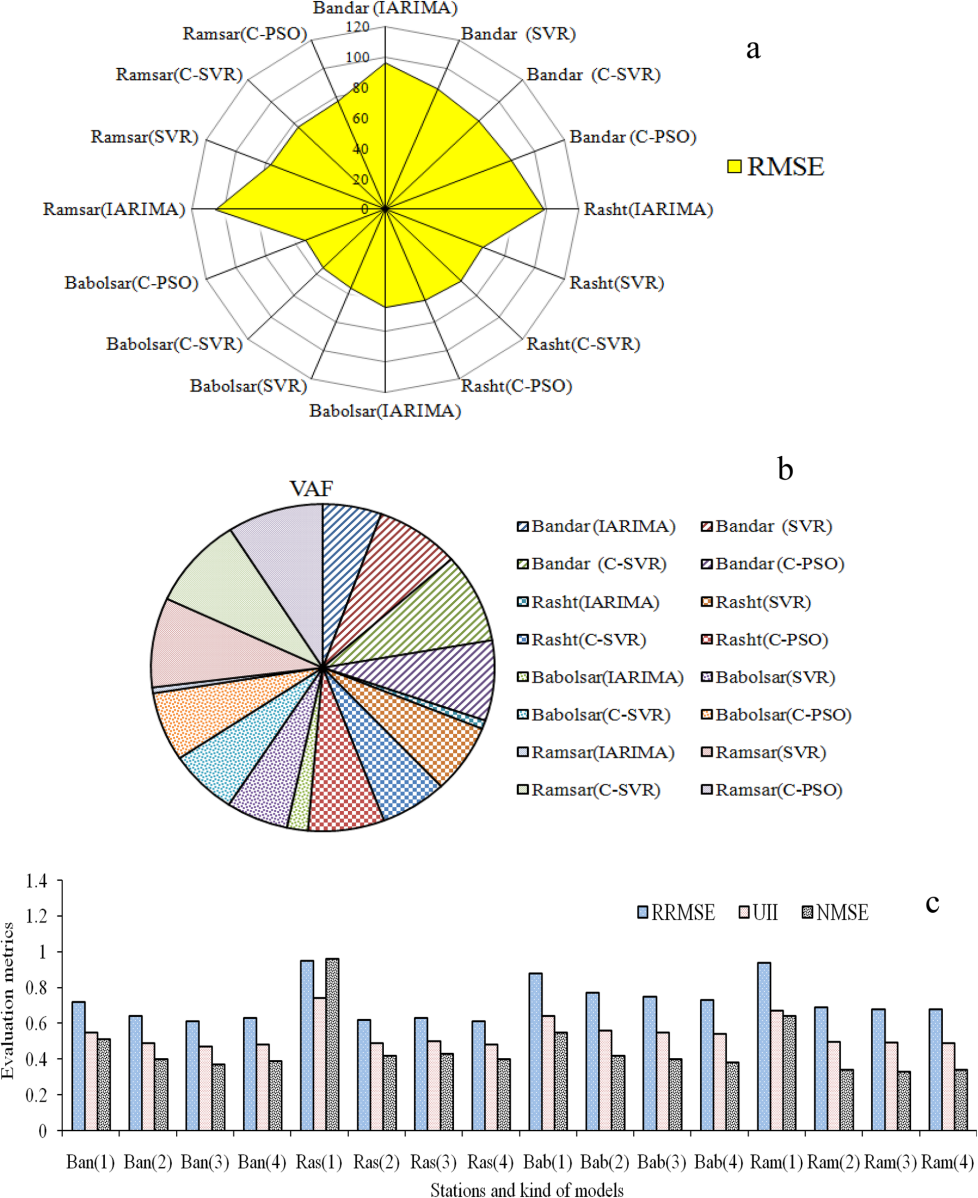
(**a**) The efficiency comparison of IARIMA and developed IARIMA using RMSE, (**b**) with VAF criterion, and **c** with RRMSE, UII and NMSE (1–2–3–4 denote the IARIMA, IARIMA-SVR, IARIMA-C-SVR, IARIMA-C-PSO and Ban, Ras, Bab and Ram stand of Bandar Anzali, Rasht, Babolsar and Ramsar) in 2017–2020.

The curves of Fig. 6 show that the similarity of observed precipitation time series is high in the IARIMA-SVR concerning the improved ARIMA. If the average of precipitation series is calculated in the validation period, the absolute difference between the observed and forecasted values is 8.56 for IARIMA and 2.96 for IARIMA-SVR at Bandar Anzali station, 12.48 and 9.04 at Rashtstation, 1.3 and 0.27 at Babolsar station and 9.93 and 3.39 at Ramsar station. Therefore, the difference between the observed and forecasted values with IARIMA-SVR is lower than IARIMA. If the average of each month is calculated in the validation period, the analysis of the obtained series, for 12 months, shows that the maximum value of precipitation belongs to November for all the models and the observation. The minimum value of precipitation is in June, May, and June for the observation, IARIMA, and IARIMA-SVR, respectively at Bandar Anzali station. The maximum value of precipitation is in October for all the models and the observation, and the minimum value of precipitation is in June, August, and May for the observation, IARIMA, and IARIMA-SVR, respectively at Ramsar station. The maximum value of precipitation is in October, September, and November for the observation, IARIMA and IARIMA-SVR, respectively, and the minimum value of precipitation is in May, November, and May for the observation, IARIMA, and IARIMA-SVR, respectively at Rasht station. The maximum value of precipitation is in October and November for the observation and used models, respectively, and the minimum value of precipitation is in May, July, and May for the observation, IARIMA, and IARIMA-SVR, respectively at Babolsarstation. The maximum matching between forecasted and observed values in terms of time correspondto the maximum values and IARIMA-SVR. Also, Bandar Anzali and Ramsarstations havethe maximum compatibility between the observation and forecasted data in the used models. The next step for development of the improved ARIMA using the forecast combination scheme is conducted in two approaches: (1) Weighting method using PSO, (2) Artificial intelligence technique. In each approach, the model sensitivity analysis has more importance in terms of the forecast values, for example with IARIMA-C-PSO, the increased RMSE with the acceleration coefficients or self/personal confidence parameter changes (from C_1_ = 2 to C_1_ = 1) is 7.84% at Bandar Anzali station. The efficiency comparison of the improved ARIMA model with the development cases is conducted using some evaluation metrics (Fig. 7).

**Figure 8:**
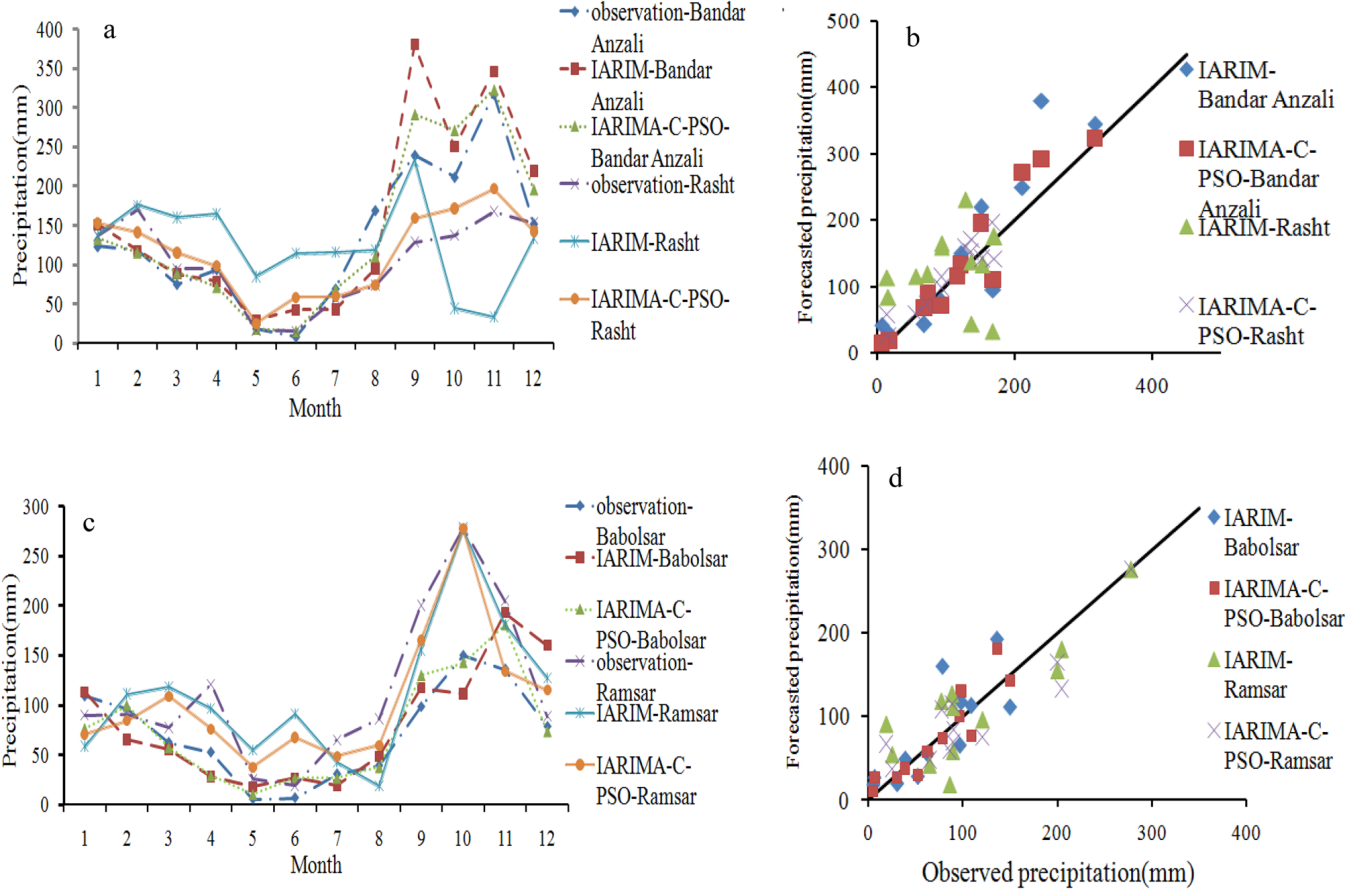
(**a**) Observed and forecasted, IARIMA and IARIMA-C-PSO, monthly precipitation time series in Rasht and Bandar Anzali, (**b**) Babolsar and Ramsar (**c**) with the scatter plot of Rasht, Bandar Anzali, and (**d**) Babolsar and Ramsar.

In general, the evaluation metrics of Fig. 7, from improved ARIMA to improved ARIMA with forecast combination scheme improved. The decrease of RMSE from improved ARIMA to IARIMA-C-PSO was 11.8%, 34.95%, 16.6%, and 27.11% at Bandar Anzali, Rasht, Babolsar and Ramsar stations, respectively. Also, the decrease in RRMSE was 12.5%, 35.78%, 17.04%, and 27.12%, and the decrease in UII was 12.72%, 35.13%, and 15.6% and the decrease in NMSE was 23.67%, 58.33%, 30.9% and 46.8% at the above-mentioned stations. Furthermore, the values of VAF increased from improved ARIMA to IARIMA-C-PSO. In contrast, the decrease of VAF values from IARIMA-C-PSO to improved ARIMA is 30.23%, 88.2%, 71.08%, and 93.46% at Bandar Anzali, Rasht, Babolsar and Ramsar stations, respectively. The VAF values in descending order belonged to the Ramsar, Bandar Anzali, Rasht, and Babolsar stations. The difference between VAF values at Bandar Anzali and Ramsar stations is low. Comparing the performance of improved ARIMA with combination concept (SVR and PSO) shows that the minimum RMSE, RRMSE, UII and NMSE values at Bandar Anzali belonged to IARIMA-C-SVR and at Rasht and Babolsar stations belonged to IARIMA-C-PSO, and the differences between evaluation metrics of IARIMA-C-PSO and IARIMA-C-SVR is low at Ramsar station. The minimum and maximum UII values of improved ARIMA with forecast combination scheme belonged to the Bandar Anzali and Babolsar stations, respectively. Among the two procedures of developed ARIMA, the second approach is superior to the first one, for example, the decrease of UII from improved ARIMA to IARIMA-SVR and IARIMA-C-PSO is 10.9% and 12.72% at Bandar Anzali station, 33.78% and 35.13% at Rasht station, 12.5% and 15.6% atBabolsar station, 25.3% and 26.86% at Ramsar station. Also, decrease of RRMSE from improved ARIMA to IARIMA-SVR and IARIMA-C-PSO is 11.11% and 12.5% at Bandar Anzali station, 34.73% and 35.78% at Rasht station, 12.5% and 17.04% at Babolsar station, 26.94% and 2712% at Ramsar station. To evaluate the performance of the developed model, the average values of months per different years were calculated and their variations are shown in Fig. 8. Also, the scatter plot is used to understand the relation between the observed and forecasted data (the plot is shown in Fig. 8).

**Figure 9:**
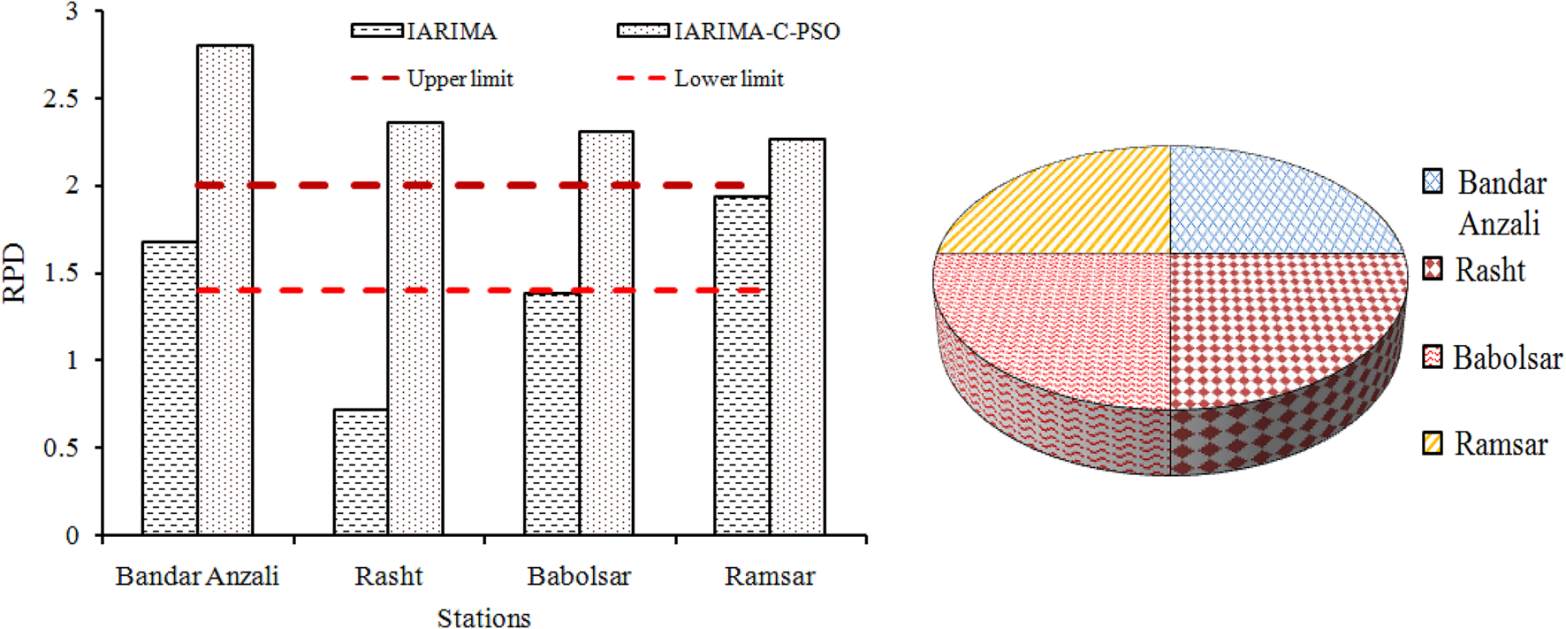
(**a**) The RPD values of IARIMA and IARIMA-C-PSO with its limits for all stations, and (**b**) the RPD average for two models of each station.

The curves of Fig. 8a–c show the similarity of monthly observed and forecasted precipitation values using IARIMA-C-PSO is higher than the forecasted precipitation values using improved ARIMA. The RMSE of all months for improved ARIMA and improved ARIMA with forecast combination scheme was 54.14 and 32.42 at Bandar Anzali station, 74.77 and 23.06 at Rasht station, 39.68 and 33.99 at Ramsar station, and finally 34.21 and 20.63 at Babolsar station. The decrease of RRMSE from IARIMA-C-PSO to improved ARIMA was 40%, 69%, 40.42%, and 14.28% at Bandar Anzali, Rasht, Babolsar and Ramsar stations, respectively. The decrease of UII from IARIMA-C-PSO to improved ARIMA is 41.17%, 69.06%, 40%, and 13.79% at Bandar Anzali, Rasht, Babolsar and Ramsar stations, respectively. The proximity of distribution points of IARIMA-C-PSO around the identity line (1:1 line) is greater than those of the improved ARIMA. The R^2^ of the fitted line in the IARIMAC-PSO is 0.92, 0.85, 0.8, and 0.83 for the Bandar Anzali, Rasht, Ramsar, and Babolsar stations, respectively. The R^2^ of fitted line decreasedfrom IARIMA-C-PSO to the improved ARIMA as 10.86% and 22.89% for Bandar Anzali and Babolsar stations, respectively. For precise comparison between the IARIMA-C-PSO and improved ARIMA, the RPD criteria was calculated which is displayed in Fig. 9.

According to Fig. 9a, the values of RPD with IARIMAatBandar Anzali, Babolsar and Ramsarstations is higher than the lower limit, and the RPD of Rasht station is below the lower limit. The IRIMA model cannot increase the RPD of forecasts greater than the upper limit, but IARIMA-C-PSO can increase the RPD of forecasts greater than the mentioned limit. The decrease of RPD from IARIMA-C-PSO to IARIMA is 40%, 69.49%, 39.82%, and 14.53% at Bandar Anzali, Rasht, Babolsar and Ramsarstations, respectively. The largest RPD of IARIMA-C-PSO corresponds to Bandar Anzali station. Calculating the average of RPD from the two models in Fig. 9b shows the contribution of Bandar Anzali and Ramsar is high.

## Discussion

The improved ARIMA is more capable of forecasting the monthly precipitation than SARIMA^2^. The factors that affect the improved ARIMA performance include (1) Accurate clustering analysis: in this part, the type of clustering analysis such as hierarchical clustering, and appropriate distance or similarity measure such as squared Euclidean distance are the effective factors. The governing climate of stations in this study is classified as humid, but the number of classes at each station with the ward method is different, and it reveals the power of clustering analysis in grouping the months with similar characteristics. (2) Perform exactly the steps of Box-Jenkins^66^ methodology for modeling the statistical characteristics of each class based on the time variation using ARIMA, for example, the increasing of Akaike information criterion (AIC) at Babolsar station from ARIMA (0,0,3) to ARIMA(3,0,0) is 27.69% for truncated mean time series of fourth class. Also, at Ramsar station, the increase of AIC from ARIMA (4,1,0) to ARIMA(1,1,3) is 14.53% for the minimum time series of the second class.

Evaluation of the improved ARIMA model at the mentioned four stations indicated the better model performance at Bandar Anzali station with a maximum correlation coefficient of (0.7), minimum RRMSE(0.68), UI(0.25), UII(0.51) and NMSE(0.47) concerning the other stations. The maximum error criteria belonged to Rasht station (for example UII = 0.78). Due to the successful performance of improved ARIMA^2,17^, the focus of this research has been on the development of the model in two procedures. The first procedure is related to the statistical characteristics modeling of each class with the associated monthly time series by SVR which had the better forecasts relative to the improved ARIMA at all stations. The decrease of RMSE from improved ARIMA to IARIMA-SVR is 12.25%, 40.5%, 13.59%, and 24.46% for Bandar Anzali, Rasht, Babolsar, and Ramsar stations, respectively. The average decrease of RRMSE and NMSE from improved ARIMA to IARIMA-SVR is 22.87% and 38.95% for all stations. The minimum RRMSE corresponded to Bandar Anzali station (0.59) using IARIMA-SVR. The higher capability of SVR concerning linear regression was proved in research works, like the accurate results of SVR concerning multiple linear regression (MLR) for the sorption capacity prediction^67^ and the better performance of SVR relative to the linear regression to develop the spatial distribution of fine particles and nitrogen dioxide^68^, accurate forecasts of SVR against those obtained by MLR to predict the removal efficiency of NI(II) ions^69^. SVR which is the extension of SVM can be used successfully for complicated regression problems^70^. SVR is based on structural risk minimization which can optimize the generalization accuracy over the training error but MLR uses the principle of empirical risk minimization and it does not consider the machine learning capability^69^.

The other procedure that is used to develop the efficiency of improved ARIMA is applying the forecast combination sceheme and it is conducted with two approaches using PSO and artificial intellegnce technique (SVR). Both approaches can incearse the effeciency of improved ARIMA such as decreased average RMSE, RRMSE, and NMSE values, and increased VAF values using IARIMA-C-PSO at all stations are 22.61%, 23.24%, 39.92%, and 70.73% concerning improved ARIMA. The comparison between the performances of used procedures in this study to develop the improved ARIMA shows the high performance of the forecast combination scheme in IARIMA relative to the IARIMA-SVR. The decreased RMSE (average of all stations) values from improved ARIMA to IARIMA-SVR and improved ARIMA to IARIMA-C-PSO are 21.24% and 23.37%, respectively and these values vary for different stations which in some stations the decreased values are greater than the average value such as Rashtstation and the decreased value is lower than the average value such as Bandar Anzali station. To reduce the errors and instability of single models, the forecast combination schemes were proposed which have superiority over the single model's performance by decreasing the model error^29,30,71^. The study of Adhikari and Agrawal^27^ on different real-world time series indicated that the forecast combination scheme performs remarkably better than the single models. A forecast combination scheme extremely decreases the risk of model selection.

One of the challenges in the forecast combination scheme is the reasonable determination of weights, which this study attempts to overcome this problem through the use of the optimization algorithm. Comparing the two approaches that were used as forecast combination schemes in this study, the performance of IARIMA-C-PSO is better than IARIMA-C-SVR. The differences between forecasts of IARIMA-C-PSO and IARIMA-C-PSO are very low at Ramsar station and at Rasht and Babolsar stations the performance of IARIMA-C-PSO is better than IARIMA-C-PSO. The forecast combination scheme aims to improve the out-of-sample prediction. The optimization algorithms is suitable with balancing between exploration and exploitation when solving the optimization probalem and they help to achive relaible results^21^. 2020). ABC and PSO algorithms were used to combine the forecats of single models in the study of Wang et al.^29^ and Wang et al.^21^ and they could increase the accuracy of forecasts against the single model's performance.

Also, the sensitivity analysis has a significant effect on the performance of two procedures used for developing the improved ARIMA such as kernel function, penalty parameter, and acceleration coefficients.

The prediction of change in direction (POCID) can measure the accuracy of the direction which has improved using IARIMA-C-PSO over the improved ARIMA, where the increase is 13.8%, 15.99%, 25.92%, and 4% at Bandar Anzali, Rasht, Babolsar and Ramsar stations, respectively. In the second approach, the improved ARIMA with the forecast combination scheme, the maximum VAF belonged to the Ramsar and Bandar Anzali stations. For better investigation, the average of forecasted months was calculated which the maximum value of precipitation at Bandar Anzali station occurred in November and it is preserved by all models (IARIMA-SVR, IARIMA-C-PSO, IARIMA-C-SVR) except the improved ARIMA. At Ramsar station, the maximum value of precipitation occurred in October and it remained so in all used models. The minimum value of precipitation at Babolsar station occurred in May and remained so in all used models. In another review, the sum of the differences between the observation and forecasted precipitations (observation-forecasting) is calculated for all months. The sign of sum for all used models at Rasht, Bandar Anzali, and Babolsar stations is negative which indicates e the overestimation, and the sign of sum at Ramsar station is positive which indicates the underestimation.

To determine the monthly variation, the RRMSE of months was calculated and the maximum RRMSE occurred in June at all the stations (RRMSE_BandarAnzali_ = 2.53; RRMSE_Rash_t = 3.12; RRMSEBabolsar = 3.39; RRMSE_Ramsar_ = 2.64) and the minimum RRMSE was 0.22 in November for Bandar Anzali station, 0.31 in January for Rasht station, 0.3 in October for Babolsar station and 0.42 in September for Ramsar station. Comparsion of the months with high RRMSE values among the stations indicates that the minimum RRMSE belonged to Bandar Anzali and then Ramsar stations, also the minimum RRMSE values in all the months belonged to Bandar Anzali station. The maximum annual observed precipitation of Bandar Anzali (1961.10 mm), Babolsar (1245.68 mm), and Ramsar (1514.80 mm) stations occurred in 2019 and remained so using IARIMA-C-PSO in the mentioned stations (1843.15 mm, 912.04 mm, 1372.03 mm). The minimum annual observed precipitation of Bandar Anzali (1217.79 mm), Rasht (911.33 mm), and Ramsar (1050.20 mm) stains occurred in 2017 and remained so using IARIMA-C-PSO at the mentioned stations (1515.06 mm, 1314.78 mm, 1043.49 mm). Another criterion that is used to investigate the performance of proposed models in this study is accuracy improvement (AI) which is defined as the RMSE difference of improved ARIMA with proposed models. Also, the AI of all proposed models was greater than zero, for example, the AI of Banadar Anzali for IARIMA-C-PSO is 0.41, which indicates that the forecasts of the proposed model are in good state and the increased trend can be observed with direction to IARIM-C-PSO. The RPD values of IARIMA-C-PSO are greater than the upper limit and that is a sign of model efficiency.

## Conclusion

The accuracy of precipitation forecasting plays an important role in water resource management and planning. Therefore, in recent years, the development of models has been researched. One of the successful models in monthly precipitation forecasting is improved ARIMA with the proof of the model capabilities using the monthly time series. Investigation of improved ARIMA structure shows the need for improvement and it is conducted in two procedures in this study. The first procedure uses the SVR for modeling statistical characteristics and monthly precipitation in each class, where the SVR improves the evaluation metrics based on the different evaluation metrics. The SVR with the structural risk minimization can overcome the deficiency of the linear regression method. Another procedure is the application of the forecast combination concept to the improved ARIMA with two approaches: (1) Wighting combination with PSO, (2) Importing the forecasts of SVR and linear regression to the intelligence model. The second procedure had the minimum error concerning the first one and this shows the power of the forecast combination principle in the optimal extraction of information in the structure of improved ARIMA, which led to the significant increase in improved ARIMA efficiency. The forecast combination takes advantage of the power of each single model. Among the two approaches of the second procedure, the weighting combination scheme with the PSO algorithm yields better results most of the time. The problem-solving process in the PSO algorithm finds the optimized weights. Therefore, the forecast combination scheme by integrating the other algorithms can improve the accuracy of forecasts. Due to the effect of optimization on the second procedure, the use of other optimization methods that are efficient and effective can impact the forecasts of improved ARIMA. The increasing number of single models that are used in the forecast combination section are effective factors for increasing the efficiency of forecasts because different models have different learning methods and the models with high efficiency can increase the accuracy of forecasts. In this study, different evaluation criteria were used, and it is noted that different criteria compare the forecasted values with observation ones considering various aspects and the coordination of a large number of criteria about a model is highly valid. Comparison of different criteria showed that the forecast of Bandar Anzali is accounted the best forecast (it has the lowest coefficient of variation between the four-time series). The proposed developed IARIMA model can be introduced as an efficient tool for monthly precipitation forecasting.

## Supplementary Information

Supplementary Information.

## Data Availability

All data generated or analyzed during this study are included in this article. Further enquiries can be directed to the corresponding author.
