# Supplementary material for: Assimilation of PSO and SVR into an improved ARIMA model for monthly precipitation forecasting
Source: Sci Rep. 2024 May 27;14:12107. doi: 10.1038/s41598-024-63046-3 (PMC11637027; doi:10.1038/s41598-024-63046-3)
Supplement: Supplementary file 1 — Supplementary Information. [file 41598_2024_63046_MOESM1_ESM.docx]

% Importing data

filename = 'monthlyprecipitation.txt';

a = importdata(filename);

t=a';

% Clustering monthly data

y=pdist(t, 'euclidean');

z=linkage(y, 'ward');

dendrogram (z)

%Writing the monthly series in a cluster

cluster1=[];

cluster2=[];

%clusternn: the last cluster

clusternn=[];

% nn=number of clusters

% s1=the number of months in the first cluster

% s2=the number of months in the second cluster

% snn=the number of months in the last cluster

nn= ;

s1=;

s2=;

snn=;

% Find the max, min, truncated mean of the first cluster

for i=1:12

for j=1:s1

Maximum (i)=max (cluster1(i,j));

end

end

for i=1:12

for j=1:s1

Minimum(i)=min(cluster1(i,j));

end

end

for i=1:12

for j=1:s1

b(j)=cluster1(i,j);

truncatedmean(i)=trimmean(b,50);

end

end

% Find the max, min, truncated mean of the second cluster

for i=1:12

for j=1:s2

Maximum (i)=max (cluster2(i,j));

end

end

for i=1:12

for j=1:s2

Minimum(i)=min(cluster2(i,j));

end

end

for i=1:12

for j=1:s2

b(j)=cluster2(i,j);

truncatedmean(i)=trimmean(b,50);

end

end

% Find the max, min, truncated mean of the last cluster

for i=1:12

for j=1:snn

Maximum (i)=max (clusternn(i,j));

end

end

for i=1:12

for j=1:snn

Minimum(i)=min(clusternn(i,j));

end

end

for i=1:12

for j=1:snn

b(j)=clustersnn(i,j);

truncatedmean(i)=trimmean(b,50);

end

end

%Build LR to model maximum, minimum and truncated mean with monthly series in first cluster

% one of the monthly series in the first cluster=y1; x1=maximum series of cluster 1, x2=minimum series of cluster 2; x3; truncated series of cluster1

%new= a new matrix with four columns: 1, x1,x2,x3;

Y1=[];new=[];

g=inv(new'*new);

kk=new'*y1;

%bb is the matrix with coefficient of multiple linear regression

bb=g*kk;

% Using ARIMA model to forecast maximum, minimum and truncated mean of each group in the validation period

% nv=validation period

nv=;

forecastedmax=[];

forecastedmin=[];

forecastedstruncted=[];

for i=1:nv

monthlr(i)=bb(1,1)+bb(2,1)* forecastedmax(i)+bb(3,1)* forecastedmin+bb(4,1)* forecastedstruncted;

end

%Build SVR to model maximum, minimum and truncated mean with monthly series in cluster 1 like linear regression; monthsvr

%Build LR to model maximum, minimum and truncated mean with monthly series in second cluster

% one of the monthly series in the second cluster=y1; x1=maximum series of cluster 1, x2=minimum series of cluster 2; x3; truncated series of cluster1

%new= a new matrix with four columns: 1, x1,x2,x3;

Y1=[];new=[];

g=inv(new'*new);

kk=new'*y1;

%bb is the matrix with coefficient of multiple linear regression

bb=g*kk;

% Using ARIMA model to forecast maximum, minimum and truncated mean of each group in the validation period

% nv=validation period

nv=;

forecastedmax=[];

forecastedmin=[];

forecastedstruncted=[];

for i=1:nv

monthlr(i)=bb(1,1)+bb(2,1)* forecastedmax(i)+bb(3,1)* forecastedmin+bb(4,1)* forecastedstruncted;

End

%Build LR to model maximum, minimum and truncated mean with monthly series in last cluster

% one of the monthly series in the slast cluster=y1; x1=maximum series of cluster 1, x2=minimum series of cluster 2; x3; truncated series of cluster1

%new= a new matrix with four columns: 1, x1,x2,x3;

Y1=[];new=[];

g=inv(new'*new);

kk=new'*y1;

%bb is the matrix with coefficient of multiple linear regression

bb=g*kk;

% Using ARIMA model to forecast maximum, minimum and truncated mean of each group in the validation period

% nv=validation period

nv=;

forecastedmax=[];

forecastedmin=[];

forecastedstruncted=[];

for i=1:nv

monthlr(i)=bb(1,1)+bb(2,1)* forecastedmax(i)+bb(3,1)* forecastedmin+bb(4,1)* forecastedstruncted;

end

%Combine the forecasts of LR and SVR models using PSO

varNum=2;

lowerB=[-100 -100];

upperB=[100 100];%

%%

particlelNum=100;

maxLoop=100;

W=1;

C1=2;

C2=2;

alpha=0.05;

%%

for i=1:particlelNum

particle(i).posation=unifrnd(lowerB,upperB);

particle(i).cost=costFunction(particle(i).posation,varNum);

particle(i).velocity=0;

localBest(i)=particle(i);

end

%

[value,index]=min([particle.cost]);

globalBest=particle(index);

%%

for it=1:maxLoop

for i=1:particlelNum

particle(i).velocity=W*particle(i).velocity+C1*rand(1,varNum).*(localBest(i).posation-particle(i).posation)+C2*rand(1,varNum).*(globalBest.posation-particle(i).posation);

particle(i).posation=particle(i).posation+particle(i).velocity;

for j=1:varNum

if particle(i).posation(j)> upperB(j)

particle(i).posation(j)=upperB(j);

end

if particle(i).posation(j)< lowerB(j)

particle(i).posation(j)=lowerB(j);

end

end

particle(i).cost=costFunction(particle(i).posation,varNum);

if particle(i).cost<localBest(i).cost

localBest(i)=particle(i);

if localBest(i).cost<globalBest.cost

globalBest=localBest(i);

end

end

end

W=W*(1-alpha);

%

bestSoFar(it)=globalBest.cost;

end

%%

disp(['best fitness = ' num2str(globalBest.cost)]);

disp(' ');

disp(['best solution found is: ' num2str( globalBest.posation)])

%

x=1:maxLoop;

plot(x,bestSoFar)

xlabel('Iteration')

ylabel('Cost')

function cost=costFunction(ww,m)

%yo: observed data in the validation period

%ylr: estimated data by linear regression in the validation period

%ysvr: estimated data by svr in the validation period

ylr=monthlr;

ysvr=monthsvr;

yo=[];

ylr=[];

ysvr=[];

error2=0;

for i=1:nv

for j=2:m

error2=error2+yo(i)-ww(1)* ylr(i)-ww(j)*ysvr(i)

end

end

end

% Comparison the efficiency of forecasted monthly precipitation by different evaluation metrics; for example RMSE

yo=[];

yend=[];

sum=0;

for i=1:nv

sum=sum+(yo(i)-yend(i))^2;

end

RMSE=sum/ nv;
